# An examination of the cognitive and affective processes in errorless motor learning: study protocol

**DOI:** 10.3389/fpsyg.2025.1685720

**Published:** 2025-11-27

**Authors:** Catherine M. Capio, William W. N. Tsang, Timothy T. T. Yam, Liis Uiga, Thomson W. L. Wong, Rich S. W. Masters

**Affiliations:** 1 Department of Physiotherapy, Hong Kong Metropolitan University, Kowloon, Hong Kong SAR, China; 2 Department of Sport and Exercise Sciences, Manchester Metropolitan University, Manchester, United Kingdom; 3 Department of Rehabilitation Sciences, The Hong Kong Polytechnic University, Kowloon, Hong Kong SAR, China; 4 School of Sport and Human Movement, Division of Health, University of Waikato, Hamilton, New Zealand

**Keywords:** motor learning, errorless motor learning, children, older adults, movement variability, perceived competence

## Abstract

Acquiring movement skills is crucial across the lifespan, supporting an individual’s participation in sports and physical activities. Errorless motor learning, which promotes success during practice, has been shown to be effective for acquiring or reacquiring (e.g., following injury/disease) movement skills. This research explores the underlying processes of errorless motor learning in children and older adults. We will investigate two distinct processes associated with motor learning: (i) cognitive processing measured by movement variability and prefrontal cortex (PFC) activity and (ii) affective processing measured by perceived competence. We will recruit two participant groups—children, and older adults—who will practice a novel throwing task in either a condition where errors are minimized or a condition where errors freely occur. We will measure task performance, movement variability, perceived competence, and PFC activity before, during, and after practice. We will control for individual cognitive abilities (processing speed and executive function) and use hierarchical linear mixed models to compare the variables between the practice conditions and to verify whether the cognitive and affective processes influence outcomes following practice. The findings are expected to contribute to our understanding of skills acquisition across the lifespan and help facilitate the (re)acquisition of motor skills by children and older adults through programs provided by physiotherapists, coaches, teachers, and health professionals. This study has been registered in the Open Science Framework Registries (https://doi.org/10.17605/OSF.IO/YTERV).

## 1 Introduction

Across the lifespan, movement skills allow us to engage with our immediate environments, engage in sports and physical activity, and maintain independent performance of daily functions. Our ability to learn and perform movement skills evolves from childhood to late adulthood (Leversen et al., 2012), and strategies to promote the acquisition of movement skills need to adapt to lifespan changes. Errorless motor learning is a strategy that has been shown to benefit children and older adults (e.g., Maxwell et al., 2017; Chauvel et al., 2012), but the effectiveness of errorless motor learning can be optimized by understanding how people of different ages learn and perform movement skills. In this study, we examine the cognitive and affective processes that occur during errorless motor learning in children and older adults.

### 1.1 Motor learning

Motor learning consists of internal processes associated with practice or experience that lead to relatively permanent changes in movement skill proficiency (Schmidt and Lee, 2020). Adams (1971) described motor learning as a problem-solving process in which movement errors are solved using cognitive resources. Thus, a cognitive process has long been considered an underlying factor in acquiring movement skills. For instance, in their seminal work, Fitts and Posner (1967) described cognitive, associative and autonomous stages of learning. During the initial cognitive stage, learners discover and use rule-based knowledge to monitor and control their movements. Movement strategies are refined during the associative stage, and subsequent performance has little reliance on rule-based knowledge during the autonomous stage. The cognitive stage has been suggested to require working memory resources to support the processing, storage, and manipulation of incoming sensory information (Maxwell et al., 2003).

With this understanding, movement skills are considered to be acquirable explicitly or implicitly (Masters, 1992). Movement skills that are learned explicitly are accompanied by the accumulation of declarative knowledge (i.e., rules, mechanics of movement) that is used to support motor performance (Maxwell et al., 2003). Movements that are learned implicitly do not necessarily require increased knowledge of rules and mechanisms to support performance, despite learners being aware that they are learning the movement(s) (Masters, 1992). For example, Maxwell et al. (2001) reported that implicit learners verbalized fewer rules related to motor performance than explicit learners did. Children have also been shown to accumulate more knowledge during explicit learning conditions (van Abswoude et al., 2020). With respect to the stages of motor learning, the cognitive phase appears particularly crucial in explicit but not in implicit motor learning. Age and intelligence (e.g., IQ), which are related to working memory capacity, have also been identified as individual factors that affect explicit, but not implicit, motor learning (Steenbergen et al., 2010).

### 1.2 Errorless motor learning

One approach to promote implicit motor learning is to minimize errors during practice, i.e., errorless motor learning. When learners experience practice errors, they seek—and test—alternative movement solutions, form rules to support movement performance, and consequently utilize working memory resources (Maxwell et al., 2001). This typically promotes explicit motor learning. In contrast, when learners experience fewer practice errors, they are less likely to utilize working memory resources to explicitly seek—and test—alternative movement solutions (Capio and Eguia, 2021). For instance, young adults learning a golf putting task actively modified their movements following unsuccessful performance, suggesting that experiencing practice errors involved hypothesis testing behaviors (Poolton et al., 2005). More recently, Banihosseini et al. (2025) reported that motor performance was unaffected by mental fatigue during errorless motor learning showing limited reliance on working memory.

Errorless motor learning promotes implicit learning by manipulating constraints, such as task difficulty. In the early stage of learning, movement training might begin with an easy task during which learners experience few practice errors (Capio and Eguia, 2021). Poolton et al. (2005) reported that early experiences of success during practice develop movement skills that tend to be independent of working memory, which is consistent with implicit motor learning. Early success in practice is critically important, and machine learning methods have shown that initial performance in skill learning strongly predicts final task performance (Abeles et al., 2023). Indeed, errorless motor learning has been consistently shown to improve movement outcomes, including gross motor skills (Capio et al., 2017) and novel sporting skills (Maxwell et al., 2017).

While freely occurring practice errors hamper performance during learning, other research has shown that performance outcomes subsequently improve significantly (Lee et al., 2016), which suggests that errorless motor learning may not necessarily be advantageous. However, a key advantage associated with errorless motor learning is that learners can manage multiple tasks while displaying effective movement performance after the learning phase. This benefit has been shown in studies of children (Capio et al., 2013a, 2013b) and older adults (Chauvel et al., 2012) and may be explained by the relative independence of errorless learning from working memory resources. For instance, children with low working memory capacity showed enhanced learning when they made fewer errors during practice (van Abswoude et al., 2015). With less reliance on cognitive resources, the apparent defining advantage of errorless motor learning lies in the ability to rely on movement skills when individuals need to engage with competing attentional demands (e.g., children in playgrounds who are socializing with others and older adults who are minding environmental constraints during exercise).

Early success in practice may also lead to advantages in motivation and self-efficacy (Lee et al., 2016). In the Optimizing Performance Through Intrinsic Motivation and Attention for Learning (OPTIMAL) theory proposed by Wulf and Lewthwaite (2016), early experiences of success increase expectancy and perceived competence, which are key drivers of affective modulation, such as increased self-confidence. As shown by Chiviacowsky et al. (2012), perceived competence and self-efficacy are associated with more effective motor learning among children. Iwatsuki and Regis (2021) also reported that among young adults who performed a throwing task with their nondominant arm, fewer errors during practice was associated with greater perceived competence and superior movement performance compared with more errors during practice. It is generally considered that motivation drives individuals to direct effort and engage with the task, thus optimizing learning (Manohar et al., 2015; Summerside et al., 2018). For instance, Capio et al. (2013a) reported that children with intellectual disability who practiced with fewer errors displayed a greater tendency to use their gross motor skills during free play than did those who practiced with more errors. Moreover, when fewer errors are associated with rewards, training has been shown to induce long-term retention of skills (Abe et al., 2011) through memory consolidation, which is evident in neural mechanisms involving dopamine pathways and the primary motor area in the brain (Zhao et al., 2024). Studies of animal models have also shown that sensorimotor cortical neuron activity concurrently represents movement performance and anticipation of rewards, suggesting neural mechanisms associated with affective modulation (Ramakrishnan et al., 2017).

### 1.3 Underlying processes of errorless motor learning

The potential underlying processes of errorless motor learning have yet to be empirically tested. Owing to the implicit nature of errorless motor learning, it has been frequently suggested that it requires relatively low cognitive demands, as proposed by Maxwell et al. (2003). For example, despite cognitive impairments, children with intellectual disability who learn via errorless motor learning display improvements in gross motor skills and the ability to perform multiple tasks (Capio et al., 2013a). In other research involving adults without impairments, learning a novel locomotor task (i.e., split-belt treadmill walking) with few errors was found to be less dependent on cognitive resources—on the basis of reaction time to a secondary cognitive task—compared to when greater movement errors were generated (Sawers et al., 2013). Earlier studies demonstrated relatively greater conscious control by learners when they experienced more errors, as evidenced by increased coactivation of brain regions associated with movement processing (Zhu et al., 2011). As processing errors to adjust movement planning requires significant cognitive resources, the efficacy of errorless motor learning could be explained by the relatively reduced cognitive requirements. However, no study thus far has offered direct evidence that such cognitive factors differentiate errorless motor learning from learning with copious amounts of practice errors during the process of skill acquisition.

A behavioral representation of the cognitive processes during motor learning is the variability of movement, or trial–to–trial deviations of movement kinematics, which become evident when task difficulties vary (Lajoie and Franks, 1997). Movement variability may be considered to represent real-time cognitive engagement that is closely coupled with motor performance. Learners display relatively low movement variability when movement tasks demand high accuracy. A study of young adults engaged in a throwing task revealed greater movement variability during errorless motor learning than during errorful motor learning, and movement variability was negatively associated with conscious control (van Ginneken et al., 2018). These findings support the proposition that errorless motor learning is associated with relatively lower conscious control.

Cognitive processes during motor learning can also be assessed through direct measures of brain activity (Nishiyori et al., 2016). However, direct measures of brain activity [e.g., electroencephalography (EEG); functional magnetic resonance imaging (fMRI)] have been limited by practical and technological constraints. For example, due to noise generated by large, dynamic body movements, the use of EEG has been limited to studies of fine motor skills (e.g., Zhu et al., 2011). Functional near-infrared spectroscopy (fNIRS), which enables monitoring of brain activity, as reflected by changes in cortical oxygenation, has been suggested to be capable of overcoming the limitations associated with large body movements (Ferrari and Quaresima, 2012). Prefrontal cortex (PFC) activity, which is considered a representation of working memory engagement (Riley and Constantinidis, 2016), may be measured via fNIRS during motor learning activity. Studies have shown that fNIRS is suitable for naturalistic and ecologically valid conditions (Ayaz et al., 2019) where dynamic motor tasks are involved (e.g., walking on a treadmill, Koenraadt et al., 2014).

Affective processes during motor learning could also explain the relative efficacy of errorless motor learning. Success during practice has been conceptualized as a mediator of the positive effect of perceived motor competence on subsequent motor performance (Li et al., 2020). Errorless motor learners could be encouraged to engage in the motor task further (i.e., more practice at their own volition), leading to skill improvements (Capio et al., 2013a). It is, therefore, possible that affective states differentiate errorless motor learning from practice conditions with freely occurring errors, where participants’ motivation for practice is enhanced by experiences of success, but this has yet to be directly examined.

Affective states in motor learning research have been measured using questionnaires. Levac and colleagues (Levac et al., 2017) used the validated Intrinsic Motivation Inventory (IMI (Ryan, 1982) to measure intrinsic motivation in a study that examined the frequency of errors and autonomy in motor learning. The IMI subscales, which consist of interest/enjoyment, perceived competence, effort, value/usefulness, pressure and tension, and perceived choice, have also been used selectively in motor learning studies that included older adults (e.g., Iwatsuki and Regis, 2021). The IMI has also been adapted to measure the perceived competence of children (e.g., Ávila et al., 2012).

### 1.4 Current study and objectives

To date, there has been no deliberate examination of the cognitive and affective processes related to errorless motor learning. From a theoretical point of view, understanding these associated processes contributes to our knowledge of skill acquisition in relation to practice errors. From a practical perspective, an understanding of associated cognitive and affective processes can guide how practice should be structured to promote more efficient learning of movement skills; in this study, specifically for children and older adults. Movement-related concerns vary across the lifespan, primarily with respect to independent and effective performance of social and occupational roles (e.g., attending school, maintaining independent mobility). Understanding the variance (or invariance) of the cognitive and affective processes of errorless motor learning in childhood and late adulthood is especially important given the need to support the design of developmentally appropriate programs during the lifespan periods when people crucially need to acquire (i.e., rapid neurodevelopment in children) or maintain (i.e., age-associated decline in older adults) the ability to move well. This current study is also complemented by related research involving young adults (see project registration at https://doi.org/10.17605/OSF.IO/ZUREH), which contributes to evidence generation across the lifespan.

This study aims to examine the cognitive and affective processes associated with errorless motor learning. We investigate whether (i) cognitive processing is reduced during errorless motor learning given its implicit nature and whether (ii) the affective state is enhanced following early experiences of success. The study involves participants in the lifespan tail ends who will participate in an experiment that contrasts errorless with errorful motor learning. We will measure task performance along with behavioral indicators of cognitive (i.e., movement variability) and affective (i.e., perceived competence) states before, during, and after the practice of a functional motor task. In addition, we will use fNIRS as a direct measure of blood oxygenation changes in the PFC, which represents working memory activity (Riley and Constantinidis, 2016).

We hypothesize that the changes during the learning phase significantly differ between errorless and errorful learning conditions for (i) movement variability, (ii) perceived competence, and (iii) PFC activity. We further hypothesize that the changes across the same variables and task performance significantly differ from before to after practice between errorless and errorful learning conditions. Finally, we will test whether the changes in task performance from before to after practice are moderated by movement variability, perceived competence, or PFC activity.

## 2 Methods and analysis

This study was reviewed and approved by the research ethics committee of the primary affiliated university of the first author (Ref. no. HE-RGC2024/NHS10). All the procedures are in compliance with the principles of the Declaration of Helsinki. Research staff will obtain written consent for participation from older adults and parents of children, while verbal assent will be obtained from the children. This study was registered in the Open Science Framework Registries (https://doi.org/10.17605/OSF.IO/YTERV). Based on the planned timeline, data gathering commenced in August 2025 and is expected to end by June 2026.

### 2.1 Participants and eligibility criteria

This study protocol was developed and reported following the updated Standard Protocol Items: Recommendations for Interventional Trials (SPIRIT) guidelines (Chan et al., 2025). The research consists of two experiments, which follow the same design but with adaptations to suit the developmental requirements of children and older adults.

Corresponding to the two experiments, the following participant groups will be formed: (i) children aged 6 to 8 years and (ii) older adults aged 60 years and older. For both participant groups, the inclusion criteria are as follows: (i) normal or corrected-to-normal visual acuity and (ii) no diagnosed neurodevelopmental, medical or orthopedic conditions that constrain the stability and mobility of the upper limb or trunk. For cognitive screening, children should not require support for special educational needs; older adults should score ≥ 24 on the Chinese version of the Mini–Mental State Examination. Owing to the task, those who are ambidextrous will also be excluded (see Procedures below for the task). We acknowledge that these exclusion criteria allow us to control the experimental conditions but limit the generalizability to populations without cognitive or physical impairments.

Using GPower 3.1.9.7 and assuming an analysis based on hierarchical linear regression, a sample size of 46 participants for each age group is needed to determine the effects of errorless and errorful learning conditions on movement performance. The parameters include an effect size of 0.23 based on improvement in task performance (i.e., number of successful hits) that differentiated errorless and errorful conditions among young adults that followed a similar protocol (Capio and Eguia, 2025), 80% power, and an alpha of 0.05. Because the procedures will take place over two sessions and fNIRS procedures may be uncomfortable and challenging to some, we will aim to recruit 52 participants per age group (i.e., ~10% additional) to mitigate the potential impact of sample attrition and/or data loss.

Recruitment of participants will be conducted by research assistants through public announcements on the social media pages of the university and nearby communities. Interested participants will be able to contact the research team directly through online messaging (i.e., WhatsApp) and will be scheduled for screening and experiment participation. Informed consent and verbal assent (for children) will be obtained by the research assistants. Within each age group, participants will be randomly assigned in equal numbers by sex to either the errorless (EL) or errorful (EF) group via stratified block randomization. The random allocation block sequence will be computer-generated by research staff not involved in participant recruitment or assignment.

Participants will be informed only about the procedures and requirements of the group to which they were allocated and without knowledge that an alternative condition exists. Due to the nature of the study procedures, research staff will be aware of group assignment for protocol implementation. Thus, while participant blinding is implemented to the extent possible, research staff cannot be blinded, and this limitation is acknowledged as inherent to the study design.

### 2.2 Study setting and procedures

The experiments will involve a seated overhand throwing task using the nondominant hand, which should render the task novel for the participants (Iwatsuki and Regis, 2021) and suitable for examining changes during the learning phase (Solum et al., 2020). All procedures will be conducted in a dedicated laboratory room with a fixed layout (i.e., equipment will be positioned in the same orientation for every participant) and free from external distractions such as noise or foot traffic. All mobile phones in the room will be in silent mode, and fluorescent lighting and temperature will remain constant throughout all the sessions. A participant will be seated on a stool at a prescribed distance from the middle of a circular target on the wall. On the basis of previous related studies, the distance to the target is 3 m for both children (Ávila et al., 2012) and older adults (Chiviacowsky et al., 2009). Our related study revealed that a distance of 4 m was suitable for young adults, who may arguably have greater physical capacity than children and older adults do (Capio and Eguia, 2025). The research staff will be trained to precisely follow a written protocol that details the standardized instructions for participants, order of task trials, rest periods, and data collection procedures. All equipment will be calibrated before each use to ensure identical settings for all participants.

**Figure 1:**
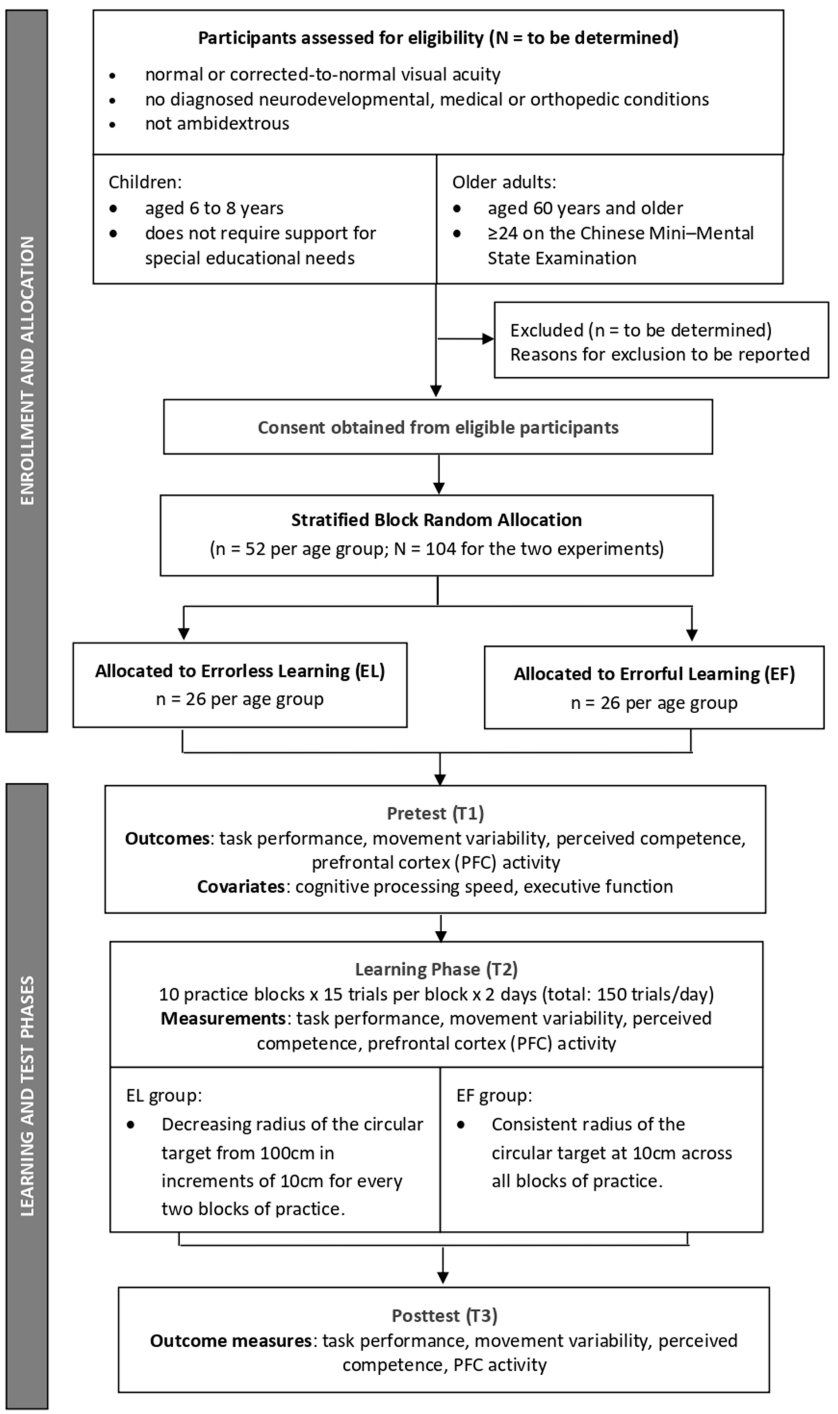
Schematic diagram of the study procedures.

Each experiment will be conducted over 2 days with 1 day in between, and it will consist of a pretest, a learning phase, and a posttest, as illustrated in Figure 1. The learning phase consists of two practice sessions. In each session, participants will perform 10 blocks of 15 practice trials per block, with two-minute rest periods between blocks (i.e., 150 practice trials per session). The occurrence of practice errors can be influenced by manipulation of task constraints, such as target size (Capio et al., 2013b; van Ginneken et al., 2018). As such, the practice conditions for the EL group consists of a circular target with a 100 cm radius which will be used in the first two blocks of practice; the target radius progressively decreases by 10 cm (i.e., 100 cm, 90 cm, 80 cm… 20 cm, 10 cm) after every two blocks. Thus, the target radius in the first practice session decreases from 100 cm to 60 cm, and in the second practice session, it decreases from 50 cm to 10 cm. The practice condition for the EF group consists of a circular target with a 10 cm radius which will be used throughout all practice sessions.

**Figure 2:**
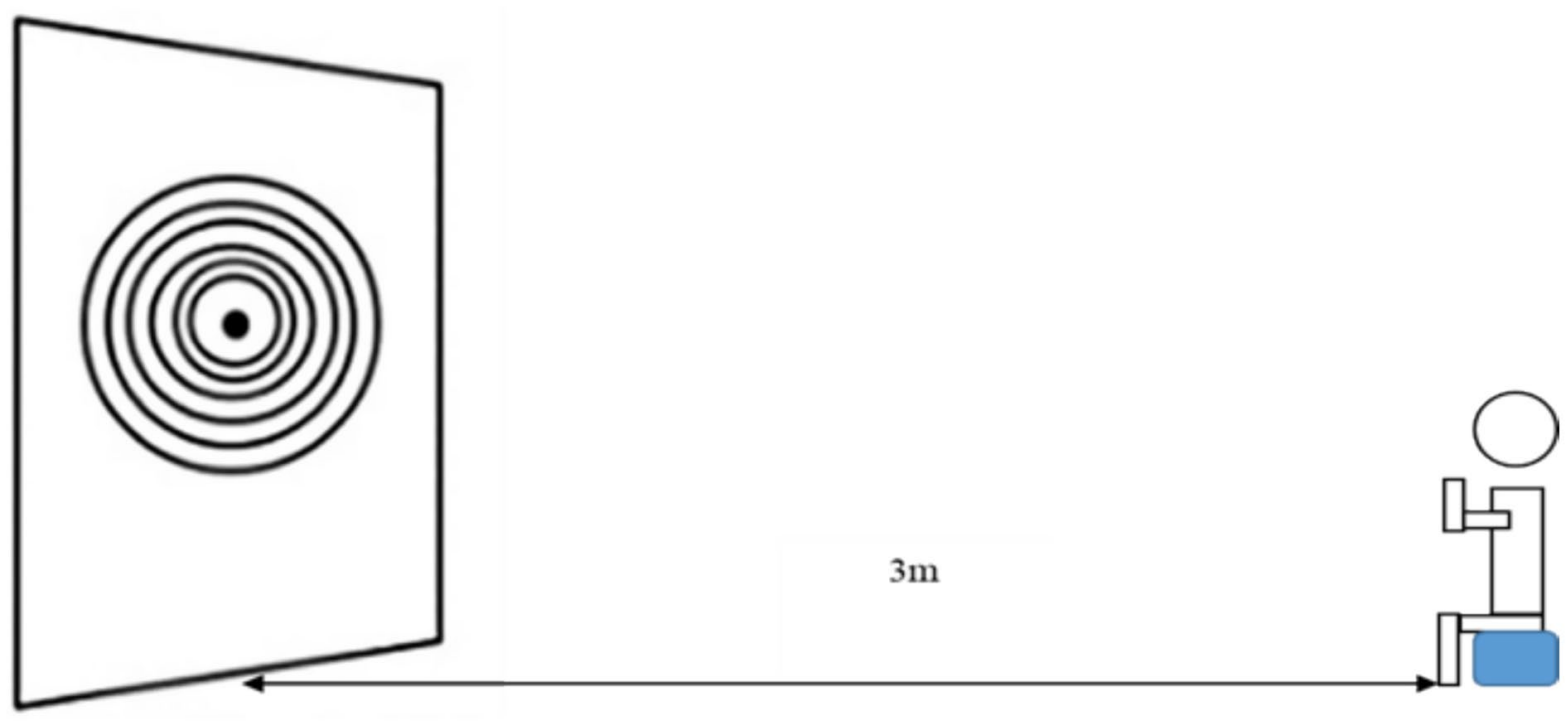
Diagram of the overhand throwing task in the test phase.

In the test phases (i.e., pretest and posttest), the target consists of 10 concentric circles with radii that increase from 10 cm to 100 cm (in increments of 10 cm). Hitting the innermost ring corresponds to the highest possible score per trial (i.e., 100 points). The score is reduced by 10 points for each ring that is incrementally more distant from the center; a score of zero is given for throws that fail to hit any part of the target. A similar task has been used in previous motor learning studies (Chiviacowsky et al., 2012). See Figure 2 for an illustration.

The pretest will be conducted on the first experimental day, immediately prior to the first practice session. It will consist of one practice trial, followed by 10 trials without any instruction or feedback (apart from being told to aim for the highest score). The posttest will be conducted on the second experimental day, immediately following the second practice session. We will adopt an A-B-A design (retention-transfer-retention) as used in motor learning studies (Maxwell et al., 2017). The first retention test will evaluate performance after the training phase; the transfer test will verify whether cognitive resources are required to support motor performance; the second retention test will assess whether any difference in performance in the transfer test was due to continued learning. The retention tests will consist of one practice trial followed by 10 trials (i.e., identical to the pretest). The transfer test consists of 10 trials with a concurrent cognitive task of counting backward (e.g., Capio et al., 2013b). The counting backward task will vary as a function of differences in cognitive ability that are associated with age. Children are asked to count backward from 100 in one (e.g., 100, 99, 98…), whereas older adults are asked to count backward from a randomly selected starting point in three (e.g., 95, 92, 89…).

Procedures for monitoring protocol adherence will include the use of attendance logs and reminders before each block to encourage engagement. We expect minimal risk of dropouts because participation will be completed within two sessions. Nevertheless, missed appointments for the second session will be followed up and rescheduled within 2 days of the original schedule. Because post-tests will be administered in the second session, participants who completely miss the said session will be excluded from data analysis. There are no requirements regarding concomitant care, but participants will be asked to report any activities that involved a similar throwing task in the day(s) intervening the two study sessions. Any deviation from the protocol, including those due to technical issues, and related concomitant care will be documented for each participant.

### 2.3 Outcome measures

The outcome measures consist of task performance, movement variability, perceived competence, and PFC activity. Covariate measures include cognitive processing speed and executive function capacity.

Task performance during the learning phase is measured by the number of successful hits on the target in each block. Task performance during the test phase is measured by accuracy scores. Task performance scores will be measured in real time by two independent observers and verified *post hoc* using video recordings of the target. The range of scores for successful hits during learning is 0–15 per block, and the ranges for accuracy during the tests are 0–100 per trial and 0–1000 for each test (Capio and Eguia, 2025).

Movement variability will be calculated using the coefficient of variation (CV) of the joint angles and joint velocity. CV is defined as the standard deviation divided by the mean (expressed as a percentage) and provides a normalized measure of how consistently joint kinematics are maintained across repeated movements (Mukherjee and Yentes, 2018). Movement kinematics will be generated using the Noraxon MyoMOTION system (Noraxon Inc., United States), which uses inertial measurement units (IMUs) to track the three-dimensional angular changes in the upper limb used for throwing. This instrument has been shown to provide valid estimates of joint kinematics (Heuvelmans et al., 2025). Four IMUs will be positioned on the (i) dorsum of the hand, (ii) dorsal side of the distal half of the forearm, (iii) lateral side of the middle third of the upper arm, and (iv) over the spine of the upper thoracic region.

Perceived competence will be measured using the corresponding IMI subscales, including adapted versions for older adults (Iwatsuki and Regis, 2021) and children (Ávila et al., 2012). The IMI uses a 7-point Likert scale (i.e., 1 = “not at all true”; 7 = “very true”). The perceived competence subscale includes statements such as “I think I am pretty good at this task,” “I was pretty skilled at this task,” and “This was a task that I could not do very well.” IMI subscales, including perceived competence, have been shown to have adequate internal consistency in the context of laboratory movement experiments (McAuley et al., 1989). While the IMI subscales have not been formally validated in our target participants, they have been used widely in motor learning studies, including those of children (Goudini et al., 2019; Levac and Lu, 2019; Drews et al., 2020) and older adults (e.g., Kappen et al., 2020; Iwatsuki and Regis, 2021; Dong et al., 2023).

PFC activity will be monitored by measurements of relative changes in oxygenated (HbO) and deoxygenated hemoglobin (HbR) levels using the Brite NIRS wireless system (Artinis Medical Systems, The Netherlands). Brite has been widely used to measure brain oxygenation in recent studies that have examined cognitive (Bhat et al., 2017) and affective (Ruotsalo et al., 2023) processes. Many studies have used the system in older adults (e.g., Goenarjo et al., 2021; Maasakkers et al., 2021; Bonnal et al., 2022) and children (e.g., Bulgarelli et al., 2023; Zheng et al., 2023; Yan et al., 2025) and have shown that it is a reliable and feasible instrument for characterizing adaptive cortical changes.

Movement kinematics and PFC activity will be measured during the pretest, across blocks three to eight in each practice session, and during the posttest. We aim to capture stable estimates of movement kinematics and PFC activity that reflect practice performance and limit the confounding effects of within-participant variability due to adaptation in the initial blocks (Eggert et al., 2021). Perceived competence will be measured after the pretest, immediately after blocks three and eight in each practice session, and after the posttest.

To account for individual cognitive processing speed and executive function capacity, we will use the Color Trails Test (CTT)-1 and -2 (Williams et al., 1995) for children and the Trail Making Test (TMT)-A and -B (Bowie and Harvey, 2006) for older adults. CTT-1 and TMT-A scores are considered measures of visual search and perceptual speed, whereas CTT-2 and TMT-B scores reflect the task-switching component of executive function. For all participants, verbal working memory will be assessed using a backward digit span test (Ramsay and Reynolds, 1995), and visuospatial working memory will be assessed using a reversed Corsi block tapping test (Corsi, 1973).

### 2.4 Safety

While two-minute rest periods between blocks are specified in the protocol across the learning and test phases, the participants will be encouraged to inform the research staff should they feel fatigue or any form of discomfort while the research staff monitor any sign of fatigue or distress. It is also acknowledged that adverse reactions may be associated with the use of Brite NIRS and IMUs. Adverse event monitoring will consist of logs maintained throughout the study, with research staff responsible for documenting any negative effects, discomfort, or unexpected responses throughout the learning and test phases. In case of discomfort or fatigue, the participants will be offered additional rest periods; they are also free to defer or withdraw their participation without any negative consequences as specified in the process of establishing informed consent. Should any unexpected findings in cortical activation or movement kinematics be observed, participants (or their guardians) will be promptly notified and referred to appropriate professionals for further evaluation as necessary. Any such adverse events or unexpected findings will be recorded, reported and followed up to ensure safety of the participants.

### 2.5 Manipulation check

Task performance during the learning phase allows us to check that the EL condition indeed had significantly fewer errors during practice, indicating adherence to the intervention. A verbal protocol will also be used for older adults, with participants asked to report rules and strategies that they used when performing the throwing task (i.e., representing accumulated declarative knowledge) at the end of each practice session. Considering the emerging verbal reporting ability of children, they will be asked to demonstrate how to perform the throwing task in a step-by-step manner.

### 2.6 Data processing and analysis

Data for movement kinematics and PFC activity will be processed and managed following proprietary standard procedures of the Noraxon MyoMOTION system (Noraxon Inc., United States) and Oxysoft (Artinis Medical Systems, The Netherlands), respectively. Data for task performance, perceived competence, and the covariates will be entered by research staff in real time via a customized data entry form on Qualtrics (Qualtrics LLC, United States). The identities of the participants will be known initially to the research team and recorded for purpose of matching the data from the two sessions. However, the link between participants’ identifying information and the data will only be made through codes (e.g., Participant 001). Once all data has been matched, all identifiable information will be deleted. All data files will be stored as password-protected files in the secure (i.e., password required) network drive of the first author’s affiliated university, with two-factor authentication enabled, and accessible only to the research team. A fully anonymized data file will subsequently be stored in an open science platform (https://doi.org/10.17605/OSF.IO/YTERV).

We will use random effects multilevel/hierarchical linear model (HLM) analysis to test our hypotheses. To test changes in the learning and test phases, we use the HLM approach to estimate growth curve models to examine change over time (i.e., practice blocks, test) on two levels—level one representing individual change over time and level two accounting for the extent to which change differs between age groups and between the EL and EF groups (Raudenbush and Bryk, 2002). The level one model will quantify individual changes in the measured variables (i.e., task performance, movement variability, perceived competence, PFC activity) as a function of time (i.e., practice block, test). Random slopes and intercepts will be specified for participants to account for individual differences in baseline performance and rate of change. For the level two model, fixed effects will include age group (i.e., children, older adults) and learning condition (i.e., EL, EF) as categorical predictors. Additional fixed effects will include the relevant covariates (i.e., cognitive processing speed and executive function). The random effects of the repeated measures estimate the relationship of the scores at the first time point to the rate of change over time. Planned interactions will include age group × learning condition to examine different effects of the learning condition across age groups and time × learning condition to test whether learning trajectories vary between the EL and EF conditions. Separate models will be fitted to each of the measured variables.

The statistical assumptions underlying HLM (i.e., normality of residuals, homoscedasticity, and independence of random effects) will be evaluated through diagnostic plots and residual analysis. If violations are detected, robust estimation methods or appropriate variable transformations will be applied. Missing data will be handled through maximum likelihood estimation under the assumption of missing at random (MAR) to retain all available data and minimize bias. Our data analysis approach is deemed appropriate because it allows us to track change over repeated measures and account for participants’ individual patterns of change by calculating estimates of within- and between-individual variation (O’Connell and McCoach, 2004).

To test whether the cognitive and affective process variables moderate the changes in movement performance from pretest to posttest, an HLM cross-level interaction model will be computed. We will determine whether the slope coefficients of movement performance vary across groups; a significant cross-level interaction will indicate that the slope of movement performance from pretest to posttest varies as a function of the moderators (i.e., cognitive and affective process variables) (Davison et al., 2002).

### 2.7 Monitoring

The first author (CMC) is the principal investigator and is responsible for the overall design, conduct, and coordination of the study including monitoring data integrity and patient safety as stipulated in the institutional ethics approval. Three co-authors (WWNT, TTTY, TWL) provides oversight of the protocol and monitors data integrity in relation to movement kinematics. Two co-authors (LU, RSWM) provides oversight of the protocol and monitors data integrity in relation to perceived competence and PFC activity. The Research Grants Council (RGC) provides financial support and exercises project monitoring that requires periodic project progress and completion reports. Amendments in the protocol will be communicated to the institutional ethics review board and the RGC. However, the RGC will not be involved in actual research processes from implementation to publication.

## 3 Discussion

This study will generate empirical data that will contribute to understanding the cognitive and affective processes associated with errorless motor learning in childhood and late adulthood. By examining these processes, this study can help increase effectiveness and promote the efficacy of errorless motor learning. The findings of this study can inform a framework based on the associated cognitive and affective processes that will identify the components of the practice environments that we can optimize. The knowledge that this study will generate also contributes to our understanding of the process of skill acquisition in relation to practice errors.

Studies suggest that there is value in applying errorless motor learning in neurorehabilitation contexts and for learners with impairments (Steenbergen et al., 2010; Kleynen et al., 2020). The findings of this study will contribute to verifying whether errorless motor learning efficacy is associated with reduced cognitive load or enhanced perceived motor competence. By examining the observed changes in movement variability, perceived competence and PFC activity, we can inform best practices in rehabilitation and motor relearning protocols. Specifically, a greater understanding of the cognitive and affective processes associated with motor learning could inform the design of interventions for skill (re)acquisition in aspects that could include optimizing feedback, structuring practice, and enhancing motivation.

Insights into the interplay of cognitive and affective indicators could inform lifespan frameworks for motor skill instruction, supporting more responsive interventions in clinical and educational settings. Given the changes in skill acquisition and movement performance from childhood to late adulthood (Leversen et al., 2012), a framework for age-appropriate skill acquisition could be developed with consideration of the cognitive and affective states of pediatric and geriatric rehabilitation clients.

### 3.1 Patient or public involvement

There was no public or patient involvement in the study design development, but the findings will be disseminated to the older adults and parents of children who will participate in this study. Knowledge from the findings will also be shared with the public via the social media platforms of the researchers’ universities. The findings will be disseminated in scientific conferences and peer-reviewed journals within the areas of psychology and human development to contribute to our understanding of skills acquisition across the lifespan. This understanding could help practitioners who facilitate the (re)acquisition of motor skills by children and older adults (e.g., physiotherapists, teachers, coaches, health professionals).

### 3.2 Strengths and limitations of this study

This study examines motor learning at the tail end of the lifespan, i.e., childhood and late adulthood, which are periods during which movement skill acquisition is critical. Behavioral indicators of cognitive and affective processes are complemented by measuring hemodynamic changes in the prefrontal cortex of the brain. The planned data analysis accounts for individual variation as participants progress through the learning and test phases. We acknowledge, however, that the study excludes those with physical or cognitive impairments, thereby limiting generalizations to populations without impairment.
